# Benchmark-Based Reference Model for Evaluating Botnet Detection Tools Driven by Traffic-Flow Analytics

**DOI:** 10.3390/s20164501

**Published:** 2020-08-12

**Authors:** Katherinne Shirley Huancayo Ramos, Marco Antonio Sotelo Monge, Jorge Maestre Vidal

**Affiliations:** 1 Faculty of Engineering and Architecture, Universidad de Lima, Avenida Javier Prado Este, 4600 Lima 33, Peru; 20112814@aloe.ulima.edu.pe; 2 Indra, Digital Labs, Av. de Bruselas, 35, Alcobendas, 28108 Madrid, Spain; jmaestre@indra.es

**Keywords:** botnet, deep learning, graph mining, malware detection, machine learning, traffic-flow

## Abstract

Botnets are some of the most recurrent cyber-threats, which take advantage of the wide heterogeneity of endpoint devices at the Edge of the emerging communication environments for enabling the malicious enforcement of fraud and other adversarial tactics, including malware, data leaks or denial of service. There have been significant research advances in the development of accurate botnet detection methods underpinned on supervised analysis but assessing the accuracy and performance of such detection methods requires a clear evaluation model in the pursuit of enforcing proper defensive strategies. In order to contribute to the mitigation of botnets, this paper introduces a novel evaluation scheme grounded on supervised machine learning algorithms that enable the detection and discrimination of different botnets families on real operational environments. The proposal relies on observing, understanding and inferring the behavior of each botnet family based on network indicators measured at flow-level. The assumed evaluation methodology contemplates six phases that allow building a detection model against botnet-related malware distributed through the network, for which five supervised classifiers were instantiated were instantiated for further comparisons—Decision Tree, Random Forest, Naive Bayes Gaussian, Support Vector Machine and K-Neighbors. The experimental validation was performed on two public datasets of real botnet traffic—CIC-AWS-2018 and ISOT HTTP Botnet. Bearing the heterogeneity of the datasets, optimizing the analysis with the Grid Search algorithm led to improve the classification results of the instantiated algorithms. An exhaustive evaluation was carried out demonstrating the adequateness of our proposal which prompted that Random Forest and Decision Tree models are the most suitable for detecting different botnet specimens among the chosen algorithms. They exhibited higher precision rates whilst analyzing a large number of samples with less processing time. The variety of testing scenarios were deeply assessed and reported to set baseline results for future benchmark analysis targeted on flow-based behavioral patterns.

## 1. Introduction

Computer systems are constantly threatened by a diversity of attackers (hackers, traitors, terrorists or even governments) who intentionally compromise the integrity, availability of confidentiality of the protected assets. Driven by different motivations, their final goal is to take advantage of specific system vulnerabilities that will enable them to conduct cyber attacks [1,2,3]. Among the most common security threats, malware infections, web related attacks, phishing, denial of service, spam and botnets are the most prevalent according to the Threat Landscape Report released by the European Union Agency for Cybersecurity (ENISA) in 2018 [4]. A botnet is a network of compromised nodes (“Bots”) connected to a centralized node (“Command and Control”) administered by a human operator (“Botmaster”) who remotely controls the Bots [5,6]. The term “botnet” is derived from the words “robot” and “network”, which evokes the Bots’ autonomy to perform several tasks. Botnets are in fact key enablers of several other cyber-attacks, hence representing one of the most serious threats in the area of network security [7,8,9]. Having this scenario in mind, organizations need to endorse the preparedness of their infrastructures in order to reactive/proactive deal with security incidents related to botnets [10], on which the emerging network management paradigms raise as promising cybersecurity enablers [11,12]. The absence of proper defensive countermeasures against them might prompt security concerns for publicly accessible systems, being that botnets have the ability to infiltrate any device connected to the Internet. The attackers lead their victims to infect their own system in order to recruit potential bots, thus harming the targeted systems. That is accomplished by taking advantage of common attack vectors such as phishing messages, host vulnerabilities exploitation, brute-forcing and related techniques [4,6,13].

Defense countermeasures against Botnet threats entail detection as their primary objective. Different approaches to detect malware have been proposed over the past years, mostly underpinned on network mining techniques, machine learning, deep learning and, in a broader sense, data-driven intrusion detection methods [14]. The literature review suggests machine-learning-based approaches as the most effective when dealing with botnet detection and, among them, the supervised analysis of behavioral patterns in network flows drives the detection of different types of botnets. Srihari et al. [15] defined pattern recognition as the ability to categorize an already identified object, characterized by a pattern into a class, a task automatically performed using classification algorithms. In the area of communication networks, pattern recognition analysis is commonly targeted on studying characteristics extracted from network traffic flows, which in the case of botnets disclose relationships between the communicating Bot and Command and Control (C&C) server, for instance, to set quarantine regions for mitigation purposes [11]. Under such classification-based threat analysis, attaining acceptable detection rates is highly tied to the configuration of hyperparameters set up for training the machine learning models. Even though some heuristic approaches might be implemented for hyperparameters estimation, more advanced methods such as Grid Search have been effectively used to boost up the performance of machine learning algorithms in the detection of botnets, as it was demonstrated by Gonzalez-Cuautle et al. [16]. In terms of classification accuracy, most of the research works on this field describe their results reasonably enough to put into perspective the adequateness of the machine learning models guided by the results obtained, thus posing a reference baseline for future research as well. However, shallow learning algorithms widely used in the literature for botnet detection [17] raise concerns regarding their accuracy since the configuration details on training, testing, validation and real predictive capabilities are sometimes overlooked. Consequently, deviations on the attained accuracy results might arise when machine-learning-based defensive schemes are implemented on real execution scenarios. Their adequateness becomes thereby arguable, particularly when tailored to botnet detection due to the lack of replicability conditions. Moreover, in a recent study, Singh et al. [18] raised the “problem of comparison” as an open challenge for effective botnet detection, stressing the inability of proper datasets and limited implementation descriptions as key constraints for which developing a proper comparative methodology is not easy but necessary to significantly improve results.

In order to provide a solid evaluation scheme aimed on proving the real effectiveness of common classifiers applied for botnet detection, in this research we perform a thorough comparison between a subset of supervised learning methods driven by a well-defined methodology. Our analytical approach examines the patterns disclosed by different botnet samples when exchanging network data from the zombie machine (bot) to the (C&C) server, defining six well-known supervised algorithms for further comparisons. Thereby, our object of study is the detection of botnets grounded on communication metrics measured at network flow level. To this outcome, we aim on developing robust machine learning models capable to deal with different botnet families for which network samples are gathered from real execution testbeds. On the other hand, performing classification with unbalanced data lead to unexpected behaviours unless a proper modeling is undergone. To tackle such limitations on reference data samples, we deepen into data splitting strategies and calibration procedures carefully examined to validate the real detection accuracy for specific botnet specimens. Our goal is also to generate well-elaborated models evidencing both their real modeling and testing accuracy and performance; for which a detailed calibration is provided. Furthermore, the Grid Search algorithm lead to optimize each of the model parameters based on the overall accuracy. It is worth stressing that our proposed methodology can also be extended to different classification algorithms or botnet families provided that the necessary network samples and flow metrics are collected. Bearing those considerations in mind, the main contributions of the presented research are listed as follows:

- In-depth literature review on malware recognition and the supervised learning methods most frequently used for botnet detection. Stressing their challenges on the current landscape as well.
- Understanding the behavior of botnets by analyzing the patterns involving the communication between bots and C&C server. Those expressed as network flow-based metrics.
- A comprehensive comparative methodology based on the selected supervised models by focusing the analysis on eight botnet families.
- Providing a detailed parameterization of the machine learning models, underpinning their optimal calibration and their testing and prediction results in the aftermath.
- An extended experimentation following the principles outlined above, along with a comprehensive discussion on the baseline accuracy achieved by similar proposals.
- Establishing a preliminary set of reference supervised algorithms with the potential of serving as benchmarking elements for further research.

The remaining of this document is structured as follows—Section 2 describes the state of the art highlighting the contributions of previous research works on malware and botnet-related detection. Section 3 describes the proposed methodology and the design of experiments aimed to validate our analysis. A detailed description of the experimental results is presented in Section 4. Then, the results discussion is elaborated in Section 5. Finally, the conclusions and future research lines are summarized in Section 6.

## 2. State of the Art

An in-depth review of the literature evidences that most of the analytical techniques for malware detection are nowadays grounded on artificial intelligence. Among the different malware specimens, botnets are the object of study in this research and the analytical approaches for their detection can be broadly categorized as those based on graph mining, machine learning and deep learning. For each, the following subsections provide a summarized vision of relevant research works stressing their advantages and potential drawbacks per evaluation scenario.

### 2.1. Graph Mining for Malware Detection

Wuchner et al. [19] proposed to employ compression-based network mining using the Subdue algorithm to find matching subnetworks with the scoring function called “Maximum data compression”. Hence, scoring the complexity of the quantitative data flow. Their research aimed to define a detection model capable of detecting malware with high accuracy. To this end, the MALICIA malware dataset was analyzed. This dataset is composed of 12 malware families such as Zeus, SpyEye and Cleaman, among others. To conduct the experiments, the Cuckoo sandbox 2 was deployed to suit the execution environment to capture the malware samples. The Subdue algorithm was used to extract unknown malware patterns and retain those that meet the level of complexity allowed by applying the Singleton pattern. Later, the Matcher classifies previously extracted malware patterns as either benign or malicious and which data can be compressed to remove matching subgroups. All patterns that were found to be a match are grouped together in the “Feature Generator” array, which detects whether the samples are benign or malicious. This approach was found to be more accurate than frequency-based network mining and, in turn, it has shown that using quantitative data streams for mining improves the quality of the mined patterns and the accuracy of the detection models.

Bou-Harb et al. [20] proposed the use of big data analysis using graph theory to identify the meeting point of botnets for subsequent mitigation. Their approach took advantage of unusable network IP addresses to identify malicious traffic on the Internet. The Carna botnet dataset (about 100 GB size) was analyzed in this study. The method involved capturing the behavior of an infected bot on an Internet scale to analyze its vulnerabilities. The statistical downward fluctuation analysis was used to distinguish between probing activities and traffic from dark net data. A second sample was made by analyzing the malicious behavior of malware specimens in a virtual environment and a spread tree model was used to isolate the bot niches. The Erdos-Renyi method was used to generate random graphs to determine the correlation between the malicious traffic and the probing traffic analyzed in the second sample. This correlation was based on entropy measurements and statistical tests to corroborate that the probing traffic was fact originated from the analyzed malware samples. Using both methods, all the nodes with a bot-edge probability, that is, similar in behavior, were removed successfully. Furthermore, it has been noticed that the latter nodes interacted more with the BotMaster and, consequently, caused a greater propagation of the botnet. The authors demonstrated as a general conclusion that the studied model provides a cost-effective network security solution to deal with botnet detection.

On the other hand, Sahu et al. [21] proposed a hybrid technique based on directed acyclic networks and Support Vector Machines (SVM) for malware detection. To carry out the experimentation, the KDD Cup 1999 dataset was analyzed. The process started performing data reduction to obtain a subset of unrelated characteristics modeling directed acyclic graphs (DAG). That is, validating that the set of characteristics exists in the DAG space. The SVM classifier was based on the Euclidean distance between the weights of its nodes and the values of the input vector. The experiment considered the types of attacks labeled in the KDD Cup 1999 dataset being them Normal, DoS (Denial of Service), Probing, U2R (User to Root) and R2L (Remote to User) attacks. At the end of the tests, the model performed correctly for the proposed task, showing higher precision and accuracy metrics with the two classification methods (ISMCS and CIDMS), having a 84% detection rate in the presence of normal traffic, 81% for DoS, 82% for Probing attacks, 85% for U2R attacks and 83% in the case of R2L attacks. Thereby, the proposed method has proven high accuracy and precision when detecting malware attacks.

### 2.2. Machine Learning Techniques

Wei et al. [22] proposed Androiddetect, a mobile application to detect malicious malware specimens under a supervised learning model. Naive Bayes and J48 decision tree algorithms were used for modeling the classifier, enabling them to analyze the behavior of malware and then categorize the samples as malicious and benign applications. A total of 219 malicious samples were analyzed, where 102 applications are read applications and the 117 applications are of unknown types as they were labeled in the virus database. At the experimentation stage, malicious applications to leak private information by sending text messages were installed. Then, the behavior of those specimens was analyzed in the search of patterns (i.e., system calls) that led to characterize the malware. At the testing phase, the analysis was performed considering 200 read applications (100 benign and 100 malicious applications) and 180 types of hybrid applications (90 benign and 90 malicious applications) and the authors demonstrated that Androiddetect performed effectively, reaching average accuracy (ACC) rates of 82.5% and 86% for Naive Bayes and J48 algorithms, respectively, and so it managed to reduce the average false positive rate (FPR) as well. Androiddetect, however, obtained a relatively low percentage in the TPR category but this issue was compensated with better results in detecting malware attacks, both in terms of ACC and TPR.

Gadelrab et al. [23] proposed a botnet detection model named BotCap based on machine learning techniques. Their research aims to address two major tasks—the in-depth analysis of network traffic packets and the gathering of information from infected hosts in the network. The collected dataset included two groups of botnets differentiated by their operation mode. The first group with Aryan, Ngr and Rxbot as botnets that communicate through an IRC channel; and the second group having Black Energy, Zeus and Vertexnet as botnets performing HTTP communications with the server. The dataset collects both benign and malicious traffic samples. J48 decision tree and Support Vector Machine (SVM) algorithms were trained to distinguish network traffic as benign and botnet. From the 55 features included in the dataset, a subset of 9 were selected as the most relevant for the analysis. Then, both algorithms were validated by computing the accuracy, recall and F1 measurement. In order to optimize the models, the Grid search algorithm was applied before performing a 5-fold cross-validation analysis. Their results showed that the proposed approach is capable of detecting individually infected hosts on a local network without the need to collect much information from the infected computers.

Yerima and Sezer [24] developed the DroidFusion framework to train a set of machine learning algorithms aimed at developing strong supervised models to detect malware attacks. To this end, four different datasets containing features extracted from two publicly available malware samples were analyzed. In the first dataset, taken from the Malgenome-215 project, cross-validation was used to extract the test data and build the DroidFusion model. The framework first evaluates the test data according to the following classification algorithms—J48 decision tree, Forest REP, Random Forest-100, Random Forest-9 and Perceptron. In relation to the first dataset, it was possible to demonstrate that the DroidFusion classification was more accurate than the base classifiers for both the malicious and benign samples with a weighted F1-score above 0.98. Regarding the second dataset, Drebin-215, the combination of supervised algorithms performed more accurately than the base classification algorithms with a F1-measurement of 0.9872, scoring slightly better than Malgenome-215. McAfee-350 was the third dataset to be analyzed with similar F-1 scoring results of 0.9788, closely followed in this case by J48 and Random Forest-100 particularly in precision when detecting malware and benign classes. Finally, testing the McAfee-100 dataset the results clearly outperformed the group of ensemble algorithms trained for this experiment, measuring a F-1 score of 0.9777. In the light of the evaluation results, it was demonstrated that DroidFusion’s combination of supervised models and calibration strategy improved the resultant accuracy and precision to detect several malware specimens.

Zhou and Pezaros [25] proposed a methodology targeting the detection of Zero-Day intrusions, but also addressing high accuracy levels for well-known malware attacks. Their proposed methodology is based on the analysis of behavioral metrics extracted from the CICFlowMeter-V3 tool with six machine learning algorithms. The CIC-AWS-2018 dataset was used to conduct training and validation of the supervised models. This dataset contains seven types of scenarios, among them botnet attacks, that have been trained with six machine learning algorithms—Random Forest, Naive Bayes, Decision Tree, Neural Networks (MLP), Discriminant Analysis and K-Neighbors. On the other hand, eight novel types of intrusions were included in the test dataset and therefore the capability of detecting zero-day intrusions is carried out. The performance of the trained models was cross-validated in terms of accuracy, recall, F1-score and overhead time. The overall results showed that most of the classifiers obtained high accuracy and F1 scores detecting the majority of network threats. In case of botnet detection, the F1 score of the classifiers showed an outstanding performance of about 1.0, with the exception of the Naive Bayes algorithm which presented the lowest performance reaching a 0.68 score. It was also shown that the Decision Tree model presented a lower overhead time in contrast to the rest of classifiers. It is to note that the Decision Tree model seems suitable for working with statistical data collected from the CICFlowMeter tool, hence prompting as a proper method to detect botnet patterns.

Alenazi et al. [26] proposed a novel detection framework designed for detecting HTTP botnet attacks. Three models are designed with this objective in mind. Firstly, the Domain Mass Detector analyzes features of DNS queries, such as the total number of queries or the number of geo locations of resolved IP addresses, associated with malicious fast flux DNS servers to recognize botnet patterns. Secondly, the Application Detector profiles host applications by scrutinizing DNS traffic requests originated by legitimate applications to be compared with suspicious profiles based on domain features such as the FQDN length, query type, among others. In addition, the Time Series Detector examines possible timing behavioral patterns of HTTP bots where scheduled communications between the C&C server and the bot have been found by analyzing inter-queries intervals. Based on this criterion, the three models carried out the detection of HTTP botnets by comparing three classifiers—Gaussian Naive Bayes, Random Forest and Decision Tree. The ISOT HTTP Botnet Dataset was used for validation, which in turn contains following HTTP botnet specimens—Blackout, Zeus, Blue, Black Energy, Zyklon, Citadel, Liphyra and Betabot. The obtained results confirmed that the Naive Bayes Gaussian model has a low performance compared to the Random Forest and Decision Tree models. On average, the Random Forest model scored above 99%, except for the Application Detector with 94.8%. The same pattern was observed for Decision Tree, whose accuracy was higher than 99%, but 95.5% when computed for the Application Detector.

Gonzalez-Cuautle et al. [16] proposed the Synthetic Minority Oversampling Technique (SMOTE) to address the difficulties to perform botnet classification in highly unbalanced datasets. The method was intended to improve the classification process with synthetically-generated balanced data, and optimally calibrating the parameters of the different ML algorithms in order to avoid overfitting. In this research work the authors analyzed two datasets—ISCX-Bot-2014, provided by the Canadian Institute for Cybersecurity with 16 different types of botnets reported; and the CIDDS-001-Coburg Intrusion Detection dataset, provided by the German University of Coburgin with multiple intrusion attacks such as port scanning, brute force and DoS; as well as benign observations are reported. The fact that significantly more benign samples than malicious ones are present, is a distinctive handicap for both datasets. To solve this unbalancing issue, the SMOTE oversampling technique was used and, as a result, the resultant minority and majority classes remain properly balanced for training five classifiers—K-nearest Neighborhoods, Support Vector Machine, Logistic Regression, Decision Tree and Random Forest. The extraction and selection of the most relevant characteristics was performed by the Principal Component Analysis (PCA) algorithm and then the Grid Search algorithm estimated the optimal hyperparameters for each classification model. Finally they evaluate the classification models using the precision, recall and F1-score metrics. The results demonstrated SMOTE + GS capability to improve the prediction of malicious samples with highly unbalanced data sets in terms of accuracy, with measurements ranging from 97.35% and 98.72% for the SVM and KNN classifiers, respectively).

A multi-layered framework for botnet detection is proposed by Khan et al. [27], where it is addressed the detection of P2P botnets. This research performs an in-depth analysis of traffic patterns inherent to P2P botnets on which machine learning classifiers can lead to categorize the malicious and normal observations. The framework encompasses four analytical layers. The first layer filters out non-P2P traffic to reduce processing overhead. For this reason, TCP control packets are selected to perform the identification without affecting the precision rate. In the second layer, P2P and non-P2P traffic is characterized by combining port filtering, DNS queries and a fast heuristic P2P identification method. Then, in the third layer, feature reduction helps in diminishing the chances of overfitting the classification model. In addition, feature extraction seeks for those features whose impact is more significant in the identification of the malicious traffic. Finally, in the fourth layer, a binary classifier categorizes P2P traffic as normal or botnet. In the experimental validation, the Decision Tree algorithm performed with a detection rate of 98.7% once applied upon the CTU and ISOT datasets, which outperformed other proposals based on Logistic Regression, Artificial Neural Networks and KNN.

### 2.3. Deep Learning Methods

Cakir and Dogdu [28] proposed a novel method of malware representation to extract the characteristics based on deep learning (word2vec). The Gradient Boosting Machine algorithm was used to classify the malware and the k-fold cross validation method was used to reduce the bias. Its goal was to achieve an accuracy rate for malware, which scored between 94% and 96%. A malware specimen dataset released by Microsoft in the year 2015 was analyzed which only considered families that contained the largest number of malware samples such as Ramnit, Lollipop, Kelihos_ver3, Vundo, Tracur, Kelihos_ver1, Obfuscator.ACY and Gatak. The samples were divided into 4 datasets to be tested independently with 5-fold cross validation. Two variables were considered for evaluation—accuracy function and log loss, none of them exceeding a 6% error rate. The results showed that the higher the malware sample size the better the accuracy rate for detecting attacks in the analyzed datasets.

The work presented by Tran et. al. [29] explored the application of a Long Short-Term Memory network (LSTM) for combating bots with Domain Generation Algorithms (DGA) for randomly creating a large number of domains from where a subset is actually used to communicate with the C&C server. The authors propose the LSTMI.MI algorithm, a novel cost-sensitive learning approach to address the class-imbalanced problem. Initially, all the abnormal domains are labeled as a single DGA class to conduct a binary classification given a domain name. Once categorized as automatically generated, the algorithm performs a second multi-class analysis to properly label the domain within the possible malicious categories. Botnet characterization is possible by extracting domain name features difficult to evade by adversaries. An extensive experimentation was performed on a dataset set fed by non-DGA (Alexa) and 37 DGA classes. As the dataset exhibits different imbalance degrees, the LSTMI.MI proven higher accuracy degrees than similar approaches. The F1-score for two-class cost-sensitive was 0.9849 for non-DGA and 0.9845 for DGA. In the multi-class scenario, an average F1-score of 0.8751 evidenced an acceptable accuracy degree, hence validating the adequateness of this supervised method in the area of botnet detection.

### 2.4. Data-Driven Intrusion Detection Approaches

Intrusion Detection Systems (IDS) are pivotal defensive elements to address the detection of diverse cyber-threats in current network deployments. They are mainly categorized in the research literature are signature-based and anomaly-based systems. Signature-based IDS requires specific patterns of malicious samples to perform detection, which raises an issue against never seen threats. On the other hand, anomaly-based IDS profile the behaviour of network traffic in order to detect deviations when no traffic categorization can be performed [30]. Different anomaly-based IDS deployments leverage data-driven learning approaches in order to detect botnets accurately, both when dealing with existing specimens and with new variants as well. However, and despite they entail widely adopted solutions, they are expected to adapt for facing the emerging challenges concerning explainability and strengthening against adversarial evasion tactics [31]. Thereby, the following related research works pave the way towards data-driven strategies on intrusion detection.

Wahab et al. [32], a dual solution to optimally distribute DDoS attack detection loads among virtual machines under a limited amount of cloud resources was proposed. The first approach allows the hypervisor to monitor the VMs activities to identify any malicious patterns and collecting suggestions from other hypervisors that had similar interactions. Monitoring and suggestions-based data were incorporated using Bayesian inference to estimate confidence scores. In addition, a resource-based confidence score simulating a play-role game between the hypervisor and the attacker was designed. It is intended to mislead the attackers who infer that some VMs are not being rigorously monitored. The results of this research showed that the dual modeling raised the detection accuracy of DDoS attacks in about 26%. In the research conducted by Abdel Wahab et al. [33] proposed a repeated game of Bayesian Stackelberg as a mechanism to detect and defend resources against simultaneous attacks of different types for cloud-based systems. His research aimed at developing a cloud system that can detect multiple types of attacks. The analysis of a Data-Driven Security (DDS) dataset contained logs of data from AWS honeypots. Once the data from the honeypots was collected a one-class SVM detector identifies abnormal activities, making it useful even with no previously registered attacks. The proposed solution outperforms the other strategies in detection performance by more than 7%. By having an optimal detection load distribution among VMs, its scalability increases compared to Collabra, which needed to analyze and monitor all the instantiated VMs. In a similar manner, Sotelo et al. [34,35] proposed a clustered-based analysis to detect anomalous scaling processes carried out in virtualized environments, laying the foundations for profiling workload-based and instantiation-based Economic Denial of Sustainability (EDoS) attacks, targeted against cloud deployments.

Li et al. [36] proposed a data-based mimetic intrusion detection game model called GLIDE as a defense against intrusion attacks for edge computing networks, combining a strategy of multi-redundancy voting algorithms to optimize their intrusion detection rate at edge computing network terminals and game theory. The model was designed to establish a measure to detect and eliminate intrusion attacks in complex environments for edge computing networks because they are characterized by having ambiguous interconnections. A comparison of their proposal with Fog-IDS and EIDS models was performed, showing that GLIDE obtained a higher performance in detecting malicious traffic with a hit rate higher than 80%. On the other hand, Ieracitano et al. [14] proposed a statistical analysis and an intelligent intrusion detection system (IDS) driven by autoencoders (AE), which was able to recognize malicious threats and ensure greater security in any public access system. The NSL-KDD data set was analyzed for experimental validation. The IDS detected multiple attack types such as DoS, R2L and Probe. The results showed that the AE50 classifier outperformed other methods with 84.21% precision in binary classification and 87% precision in multi-classification.

### 2.5. Research Gaps on Comparative Assessment

The four approaches reviewed in this research have exhibited a variety of applications of supervised learning to conduct botnet detection even when their modus operandi differ substantially. In particular, machine learning methods such as Decision Trees, have embraced a wide range of detection scenarios where their effectiveness has been demonstrated. However, it is to bear in mind the comparison challenges [18] stated in Section 1 emerge when dealing with unbalanced data samples, weak feature extraction and/or selection procedures, scarce description of the supervised models parameterization, or a combination of those factors, pose major limitations to develop accurate botnet detection models. Thereby, the main purpose of our research lies in filling such methodological gap for comparative purposes and so conducting a fine-grained evaluation scheme by instantiating a subset of the most effective ML techniques for a thorough measurement of accuracy metrics when detecting botnets.

## 3. Methodology

This section describes the research methodology, the description of datasets, flow metric measurements, feature selection and machine learning model construction.

### 3.1. Processing Stages

**Figure 1:**
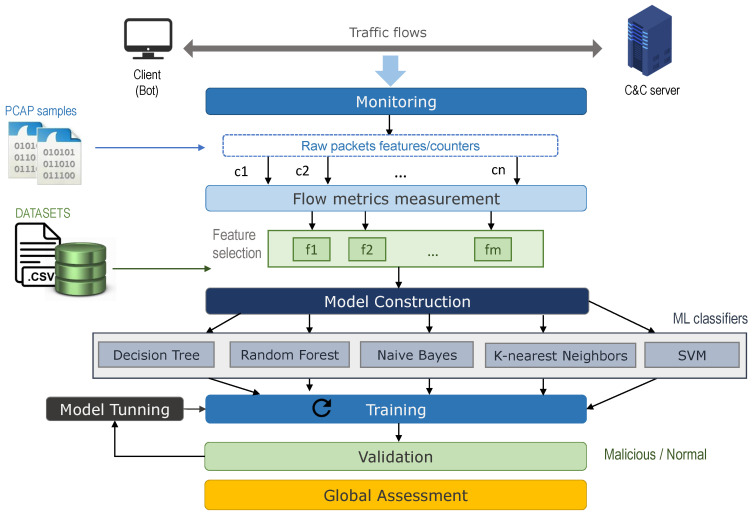
Communication scheme for metrics selection and model comparison.

The analysis of traffic-flow patterns targeted on observing, understanding and characterizing the behavior of botnets at the network level is the object of study in this research. To illustrate the communication scheme and the performed analysis of such network traffic-flow patterns, Figure 1 depicts the client-server architecture where botnet traffic is exchanged from the compromised machine (bot) to the Command and Control (C&C) server. It is to note that regardless of the number of machine learning methods considered in this proposal, they can be easily extended to further algorithms as our methodology remains generic. Grounded on the botnet analysis conducted on similar proposals [22,23,24], and to address the proper characterization of this variant of malware, five processing stages have been defined—collecting network traffic samples (benign and malicious), flow metrics measurement, implementation of machine learning models, training, validation and prediction assessment. They are explained in the following subsections.

#### 3.1.1. Collecting Network Traffic Samples

At this stage, traffic samples are obtained from different botnet specimens captured on the network, as well as normal traffic observations. These samples are commonly gathered with traffic monitoring tools (e.g., Wireshark, Tcpdump), and the captured network packets are exported using standardized formats such as PCAP. Similarly, malware datasets of raw network traffic are represented; or in some cases aggregated metrics are already processed from the captured samples either at the packet or flow level. This stage of the methodology opens the possibility for deploying monitoring tools to obtain traffic captures in a controlled environment or collect them from available datasets. For experimental purposes, the latter consideration has been assumed in this research since our goal is to strengthen the comparison.

#### 3.1.2. Flow Metrics Measurement

Given a collection of network packets, relevant traffic-flow metrics are measured to analyze common traffic patterns that characterize the behavior of different botnet specimens. Network flow metrics typically represent quantitative relationships such as—the number of packets transmitted from the source IP address 192.168.50.31 to the destination 192.168.50.88 in the forward direction is 3, with one packet going backward. Flow-level metrics have been extensively used to model data-driven detection models to tackle with various network threats, having defensive solutions against DDoS as one of the most recurrent applications in the literature [37].

#### 3.1.3. Model Implementation

In this phase the detection model is built with the selected classifiers. As a result of the literature review, some of the most prevalent methods for botnet detection have been chosen—Decision Tree, Random Forest, Naive Bayes, K-Nearest Neighbors and Support Vector Machines (SVM).

#### 3.1.4. Training

At this stage, the models are trained using a subset of the dataset. The training approaches and the tuning processes applied for each machine learning method are detailed later in this section.

#### 3.1.5. Validation

The results of each detection model are evaluated following a three-step process—(1) setting the training results as a baseline, (2) performing a cross-validation analysis on the training data set for each botnet family, and (3) assessing the botnet prediction accuracy using the test data set.

#### 3.1.6. Global Assessment

Based on the detection capabilities previously acquitted, an in-depth analysis is carried out, putting into perspective the adequacy of the detection methods for each botnet type.

### 3.2. Datasets of Botnet Traffic

In order to conduct the experimental validation of this proposal, two publicly available datasets have been used in this research:

#### 3.2.1. Cse-Cic-Ids2018 Dataset

This dataset is the result of a collaborative project between the Communications Security Establishment (CSE) and the Canadian Institute for Cybersecurity (CIC) [38]. This dataset collects daily traffic samples composed by benign and intrusion data captured in a virtualized environment. The dataset includes raw network packets (in .pcap format) and Windows/Ubuntu log files monitored on each client node. In addition, the dataset provides 80 network flow-traffic features extracted with CICFlowMeter-V3 (in .csv format) on a per-machine basis, thus providing flow-based statistical information. The different attacks simulated in the same dataset were—brute force, Botnet, DoS, DDoS, web attacks and infiltration of the network from inside. For each type of attack, network topology has been deployed in a private AWS cloud intrusion [39,40,41]. As this research is focused on the study of botnet attacks, we have analyzed traffic samples from two different botnet families—Zeus and Ares.

#### 3.2.2. Isot Http Botnet Dataset

This dataset is the result of a research work of Alenazi et al. [26] and differentiates two broader categories. The first, provides a botnet dataset generated by capturing malicious DNS traffic only, whereas the second includes benign traffic obtained after capturing legitimate DNS traffic generated by different software applications such as antivirus, online chat and instant messaging applications (e.g., Skype, Facebook, Messenger), among others. This information was collected to develop a virtual environment used to implement different kits of exploits both for HTTP botnets and legitimate software applications. There, nine command and control (C&C) servers were implemented, one for each botnet type. Domain names were configured for the C&C servers with the purpose of monitoring the behavior of outgoing DNS queries from the client nodes. For instance, the Citadel botnet C&C server domain name was registered as citadel.botnet.isot. The IP/name distribution of bots within the virtual environment was—192.168.50.14 for zyklon.botnet.isot, 192.168.50.15 for blue.botnet.isot, 192.168.50.16 for liphyra.botnet.isot, 192.168.50.17 for betabot.botnet.isot, 192.168.50. 18 for blackout.botnet.isot, 192.168.50.30 for citadel.botnet.isot, 192.168.50.31 for citadel.botnet.isot, 192.168.50.32 for be.botnet.isot (Black energy) and 192.168.50.34 for zeus.botnet.isot [42].

### 3.3. Flow-Metrics Measurement

The CIC-AWS-2018 flow features are already represented in csv format, so no additional data transformation was required. However, as the ISOT HTTP Botnet Dataset is composed of five pcaps files, they were processed through the CICFlowMeter tool. This application allowed the generation of csv files with 84 flow-based metrics. For each report, the different types of exploits were labeled using the Wireshark tool and only those records referred to botnets were preserved. Upon the five obtained reports, it was decided to work with the fourth one as it contained samples of the 8 botnets families in a time frame elapsing from 1 June to 3 June 2017.

### 3.4. Feature Selection

The following considerations have been assumed for feature selection.

#### 3.4.1. Behavioral and Contextual Features

From the total number of flow-based attributes obtained in the previous step, two groups of features were selected in accordance with previous research. The first group of eight characteristics was chosen following the methodology introduced by Sharafaldin et al. [41], where a Random Forest Regressor was used to obtain the behavioral metrics (Subflow_Fwd_Byts, Subflow_Bwd_Byts, TotLen_Fwd_Pkts, TotLen_Bwd_Pkts, Fwd_Pkt_Len_Mean, Bwd_Pkt_Len_Mean, Fwd_Pkts/s and Bwd_Pkts/s) described in Table 1. The second group of nine features was added based on the methodology presented by Gonzalez-Cuautle et al. [16], where the ISOT HTTP Botnet Dataset was used ( Src_Port, Dst_Port, Flow_Duration, Flow_Byts/s, Flow_Pkts/s, Tot_Fwd_Pkts, Tot_Bwd_ Pkts, Subflow_Bwd_Pkts and Subflow_Fwd_Pkts). 2 features were selected using the ‘feature_importances’ criterion to improve the precision of the Random Forest algorithm—Fwd Pkt Len Max and Fwd Pkt Len Min. Finally, 2 features were added to own criteria to provide more information—“Protocol” and “Label”. Consequently, the CIC-AWS-2018 dataset has 19 input variables and one output variable; whereas the ISOT HTTP Botnet dataset has 20 input variables and one output variable. This is because the “Src_Port” attribute was not found the CIC-AWS-2018 dataset. The name of the variable “Label” has been changed to “Output”. These features are described in Table 1.

#### 3.4.2. Exploratory Data Analysis

**Figure 2:**
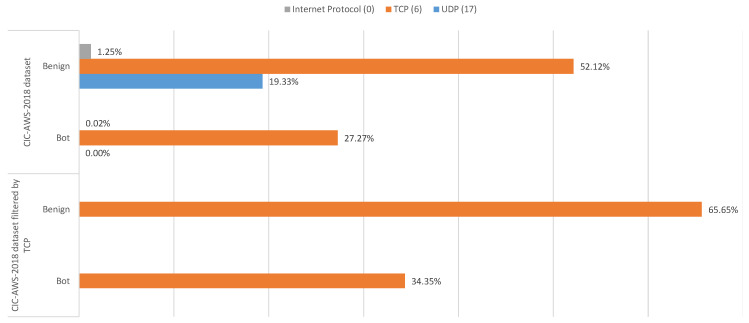
Distribution of benign and malicious traffic samples in the CIC-AWS-2018 dataset.

To get a general idea of the CIC-AWS-2018 distribution, a bar chart was plotted for each feature in contrast to the class attribute as shown in Figure 2. It can be noticed a concentration of botnet samples with 27.27% for the TCP (Transmission Control Protocol) protocol. The destination ports associated with this protocol are 8080 and ephemeral ports, a range of TCP ports that require a number of auxiliary ports to communicate with other machines [43]. Because the number of benign samples is highly concentrated on TCP traffic, the entire dataset was filtered by TCP. Hence, the portion of botnet samples has risen to 34.35%.

**Figure 3:**
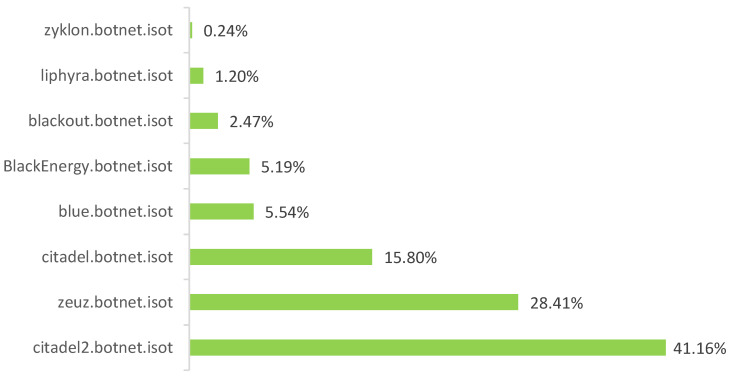
Distribution of botnet traffic samples grouped by class in the ISOT HTTP Botnet dataset.

When the ISOT HTTP Botnet dataset was analyzed, it was found that the majority of flows were transmitted using the UDP protocol (17). Additionally, a comparative analysis between botnet types was carried out to find out the concentration of them across the dataset. The result of this analysis is shown in Figure 3, where it can be seen that the Citadel botnet represents the highest percentage of samples (41.16%) and the Zyklon botnet presents the lowest proportion (0.24%) of the dataset.

### 3.5. Machine Learning Models Construction

The main goal is to identify whether the information received in the flow belongs to benign or malicious traffic. This being the main target of supervised classification, the objective is to find the best classification model that fits both datasets. Since the proposal introduced a novel reference model for traffic-flow based botnet detection, they are expected to serve as preliminarily benchmarking elements for further applications, thus needing to fulfill the following requirements—(1) they must be early-adopted solutions; (2) their pros/cons shall be well understood by the research community; and (3) there is a large variety of adaptations/modifications in the state-of-the-art solutions; and (4) to outperform them is viable, so they motivate their comparison against novel proposals. To this end, exhaustive tests are carried out with the most prevalent machine learning classification models for botnet detection documented in the research literature, as it is supported by Alenazi et al. [26], Gonzalez-Cuautle et al. [16] and Zhou & Pezaros [25]. Those classifiers are—Decision Tree, Naive Bayes, Random Forest, K-Nearest Neighbors and Support Vector Machine (SVM). It is to bear in mind that such selection fits also with the data-driven IDS based on anomaly detection as pointed by Apruzzese et al. [17] in a deep analysis of the family of algorithms best suited for botnet detection. For all cases, the datasets are splitted as 80% for training the model and 20% for testing. Some considerations have been assumed for each classifier, as explained in the following subsections:

#### 3.5.1. Decision Tree Implementation

The maximum number of characteristics depends on the number of input variables in the dataset. The minimum amount of sample size required to divide the internal node equals 2 and to be in the leaf node equals 1. To define the maximum tree depth level, a fit chart was used for both datasets. For the ISOT HTTP Botnet dataset the model was created with a depth level of 51, and for the CIC-AWS-2018 dataset it was observed that a high accuracy rate was obtained with a depth level of 14.

#### 3.5.2. Gaussian Naive Bayes Implementation

Given the simplicity of this method, the parameter that interprets the variance of all features was set to $1\times 10^{-10}$.

#### 3.5.3. Random Forest Implementation

In this model, the number of trees built from 12 and the maximum number of features depends on the number of input variables in the dataset. The minimum number of the samples required to split is 2, and the minimum number of the samples to be at leaf node is 1. Upon this parameterization, the maximum tree depth is defined using a fit chart for both datasets. For the ISOT HTTP Botnet Dataset the model is built with a depth level of 35 and for the CIC-AWS 2018 dataset it is observed that with a depth level of 22 a high accuracy rate is obtained.

#### 3.5.4. K-Nearest Neighbors Implementation

For the implementation of this algorithm, it was decided to use the Euclidean distance and the leaf size is kept as 30 by default. To find the nearest K neighbor, Gonzalez-Cuautle et al. [16] in their research implemented a Grid Search algorithm for defining one of their parameters resulting of this way three algorithms, which are Ball tree, KD tree and Brute Force. Taking into consideration these three algorithms, it was observed that the only algorithm that correctly predicted all classes was KD tree with a value of n_neigbors = 1 for the ISOT HTTP Botnet Dataset and with a value of n_neighbors = 2 for the CIC-AWS-2018 dataset.

#### 3.5.5. Support Vector Machine Implementation

The OneVsRestClassifier Multiclass was used to predict several classes. LinearSVC was used for the construction of the model because it better suits large datasets [44]. It was decided not to use dual optimization since the number of samples exceeds the number of features contained in both datasets. The parameters that define the tolerance (tol) and the adjustment parameter (C) were obtained by heuristics, so the tolerance was defined with $1\times 10^{-10}$ and C equal to 175. The maximum number of iterations is 12,000.

#### 3.5.6. Grid Search Implementation

Using the features already defined in Table 1, the implementation of each of the models was explained in the previous subsection and is also visually defined in Appendix A, which shows the list of parameters used in each of the models. In this appendix, the CIC-AWS-2018 dataset was filtered by the TCP protocol and the ISOT HTTP Botnet Dataset was divided into minority and majority classes to balance both datasets. After dividing the ISOT HTTP Botnet Dataset into majority and minority classes, the parameter which estimates the weights of the classes for the unbalanced data sets called “class_weight” was modified in the Random Forest, Decision Tree and SVM models configuration as “balanced” because it allowed to replicate the smaller class until it had as many samples as the larger one, but implicitly.

In order to improve the performance of each of the models, the Grid Search algorithm was used. For this purpose, a range of possible values was defined for each parameter in the different ML models, which are indicated in Appendix B. The result of this configuration is shown in Table 2, where the best hyperparameters per machine learning model are detailed.

### 3.6. Execution Environment

The experimental validation was developed in Jupyter with Python 3.3.2. The Scipy, Scikit-learn [44] and Pandas machine libraries were used to implement the detection models. The experiments were run in a Windows 10 host with 8GB RAM and a 2.8 GHz Core i7 processor. The DecisionTreeClassifier method was used in the implementation of decision trees and the RandomForestClassifier method was used for ensemble models. Likewise, the Gaussian method was used for the Naive Bayes model whereas the LinearSupportVectorClassifier method was used for implementing the SVM model. Finally, the KNeighborsClassifier method was used in the case of Nearest Neighbor models.

## 4. Results

This section provides a detailed explanation of the experimental results obtained in this research.

### 4.1. Cic-Aws-2018 Dataset

The CIC-AWS-2018 test dataset was evaluated with the following supervised learning classifiers—Random Forest, Decision Tree, Naive Bayes Gaussian, Support Vector Machine (SVM) and K-nearest neighbours. After analyzing the original CIC-AWS-2018 dataset, it was found that the Random Forest model turned out to be the most appropriate to correctly predict a botnet with 99.998% of precision over the rest of the models with a slight difference, with the exception of the Naive Bayes model (43.831% of precision). However, in spite of the average accuracy exceeding 94%, the results are biased, so it was necessary to balance the data. For this reason, the CIC-AWS-2018 dataset was filtered by TCP, being the predominant transport protocol as it is shown in Figure 2, where most botnets are grouped. Table 3 and Table 4 show the higher precision obtained by filtering the CIC-AWS-2018. It is to note that the detection accuracy broadly increases after implementing the Grid Search algorithm in each of the evaluated models. Based on these results, it is seen that Naïve Bayes obtains the lowest performance compared to other classifiers.

### 4.2. Isot Http Botnet Dataset

The results of the ISOT HTTP Botnet Dataset are shown in Table 5 and Table 6. There, the precision results of the ML models, before and after implementing the Grid Search algorithm, are detailed. It is observed that the GS-based models increase their accuracy, precision and recall rate. This case was observed when all models increased a precision rate of 100%, except for KNN which performed over 93%, to detect the Blackout botnet. Only the Random Forest and Decision Tree models were able to detect a high percentage of precision but a low percentage of recall to the Liphyra botnet. Compared with Decision Tree and Random Forest models, KNN reduced its precision rate for the majority of the botnets, as is the case of Liphyra. The Naive Bayes obtained a low percentage of precision but a high recall rate when analyzing Liphyra. In the case of the Citadel botnet, it was observed that all algorithms correctly detected this specimen in a virtual machine with an IP 192.168.50.31 but its precision significantly lowers when evaluated in the virtual machine with IP 192.168.50.30. Based on these results, it is possible to remark that the detection of the Citadel botnet might be influenced by the virtual execution environment.

As the Zyklon botnet contains fewer samples in the dataset, its analysis exhibited that Random Forest and Decision Tree models are the only ones capable of obtaining a high precision but a low percentage of recall. On the other hand, the Naive Bayes and SVM models obtained a low percentage of both precision and recall when analyzing the Zyklon and Blue specimens, thus failing to correctly classify those samples. Likewise, SVM were unable to classify the Liphyra botnet as well. Again, these results are explained due to the fact that there is a shortage of samples in of these specimens in the dataset as noted in Figure 3.

Because all the evaluated models improved after implementing GS, in terms of accuracy, precision and recall, it was decided to divide the ISOT HTTP Botnet Dataset into minority and majority classes to balance the data. The results of this analysis are shown in Table 7, where it is indicated that the Blackout, Blue, Liphyra, Black Energy and Zyklon botnets belong to the minority class, being the rest part of the majority class. Naive Bayes increased its precision and recall and successfully managed this class but obtained a low precision and recall rate to detect the Zyklon botnet. It can also be observed that SVM and KNN did not reach at most 80% in precision to detect the Zyklon botnet; being Random Forest and Decision Tree the models that obtained a high precision in classifying this specimen correctly. For the Zeus botnet, the Random Forest and Decision Tree models proved to handle this class perfectly; and the K-Nearest Neighbors and SVM models slightly reduced their precision and recall rate. It is also notable that GS-based Naive Bayes obtained a high precision rate and a low recall rate. On the other hand, almost all models showed a low precision and a high recall rate for detecting the Citadel botnet. The models are detecting the class well but seem to include samples from other classes, which makes their detection a little more complex. However, this was not the case when detecting the Citadel 2 botnet, since all models computed high precision and low recall rates. The models have slight difficulties in recognizing and identifying this class, but despite this issue they performed well in general.

### 4.3. Isot Http Botnet and Cic-Aws-2018 Comparison

A comparative analysis was performed between CIC-AWS-2018 and ISOT HTTP Botnet datasets in terms of accuracy as shown in Table 8. Naïve Bayes had the lowest performance among the trained algorithms (below 50% in classification) after analyzing the ISOT HTTP Botnet. The Decision Tree classifier with a depth value of 10 led to the creation of the best tree with an average accuracy of 78.893%. Since the Random Forest model is derived from Decision Tree, it was observed that with setting a depth level of 20 the Random Forest model obtained the highest accuracy rate of 79.115%, thus outperforming the rest of the classifiers. For the CIC-AWS-2018 dataset filtered by TCP, the Random Forest and Decision Tree models scored an accuracy rate of 99.999%, exceeding K-Nearest Neighbors by a small difference. Naive Bayes obtained the lowest accuracy rate of 90.901% as the algorithm modeled a binary classifier where the leaves were composed of only two samples (bot and benign) and only one tree. After splitting the ISOT HTTP Botnet Dataset, it was remarkable that Naive Bayes improved its accuracy in detecting the minority classes, scoring higher than 95%; but decreased to 56.266% when detecting the majority classes. Nonetheless, this result was significantly higher when compared to the original dataset. Likewise, there is also an improvement in the SVM accuracy as the original dataset predicted correctly few classes, but after partitioning the accuracy rate considerably improved.

**Figure 4:**
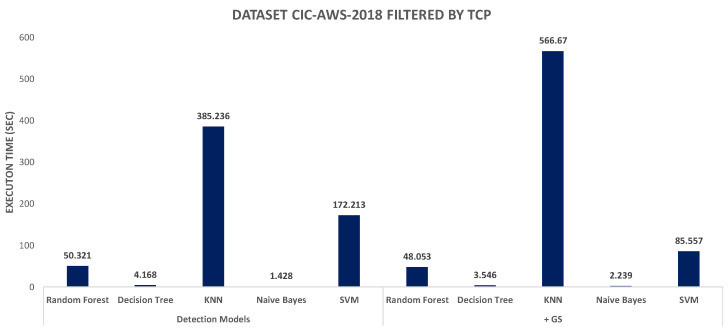
Execution time in different models using CIC-AWS-2018 dataset filtered by TCP.

**Figure 5:**
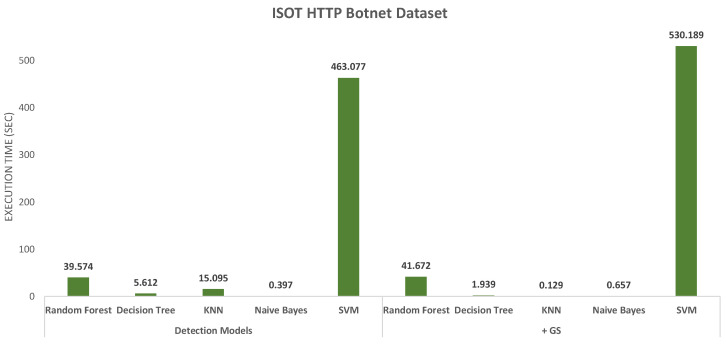
Execution time in different models using ISOT HTTP Botnet dataset.

**Figure 6:**
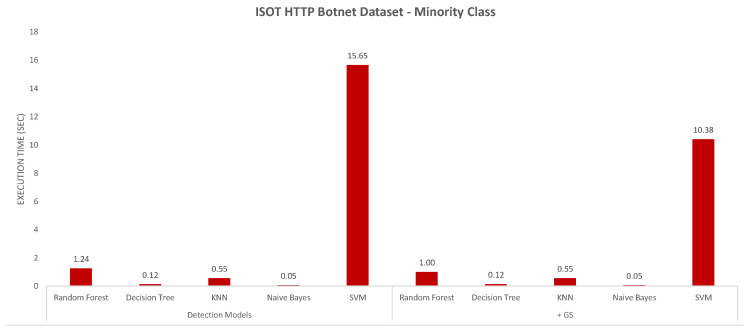
Execution time in the different models with minority class on the ISOT HTTP Botnet dataset.

**Figure 7:**
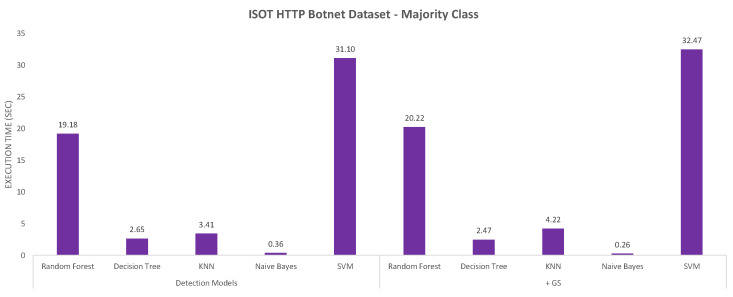
Execution time in the different models with majority class on the ISOT HTTP Botnet dataset.

The next evaluation criterion was the execution time measured for the different models, which are charted in Figure 4 and Figure 5. For the CIC-AWS-2018 dataset (Figure 4), the highest processing overhead, before and after combined with Grid Search, was generated by KNN. On the opposite, the models with less execution time are Naive Bayes and Decision Tree. As reviewed above, the Naive Bayes model shows imprecise results when detecting different botnet samples and is also the least accurate model. The execution time measured for the ISOT HTTP Botnet dataset (Figure 5) shown, in contrast to the first analysis, the highest execution time with SVM. The same indicator was measured for the minority and majority-class analysis of the dataset, and their results are shown in Figure 6 and Figure 7, respectively. In both scenarios, SVM had the least performance with execution times of 15.65 and 10.38 s, which considerably exceeded the other classifiers.

### 4.4. Cross-Validation Results

A 5-fold cross-validation was considered to check the models’ accuracy. Table 9 details the measurements obtained by analyzing the CIC-AWS-2018 dataset and the ISOT HTTP Botnet dataset. Average accuracy rates above 90.236% were calculated in the CIC-AWS-2018 dataset with minimal variances between the base model and the GS refinement. On the other hand, the accuracy variations in the ISOT HTTP Botnet dataset are more visible but exhibit the pattern mentioned in the previous section, having Naive Bayes and SVM as the least scoring methods.

### 4.5. Prediction Results

For both datasets, using the features already defined in Table 1, the construction of the models was carried out. The parameters defined for each models without GS are shown in Appendix A whereas Appendix B details the parameter calibration after GS optimization. For each model, a range of possible values was defined in order to find a set of hyper-parameters that optimize model performance. Finally, Table 2 shows the final results using GS, and it also summarizes the list of parameters used in the implementation. Thus, the prediction results for the different datasets were obtained.

Prediction tests were performed for the models before and after implementing the Grid Search algorithm on the CIC-AWS-2018 dataset, and the results obtained are shown in Table 10. It is noted that Decision Tree, Random Forest and K-Nearest Neighbors models correctly predicted the samples and were also able to recognize a benign sample or bot with 100% probability. However, after refining their models with GS, Naive Bayes was not correctly predicting bot samples; which indicates the existence of samples that have been classified as benign instead of bot. The same applies for the SVM algorithm regardless of the GS calibration.

Prediction tests were also performed for the ISOT HTTP Botnet dataset as shown in Table 11. There, the Decision Tree model correctly predicted all the samples with a 100% rate. This result might warn a possible model overfitting, but this situation was further validated by running the Grid Search algorithm to optimize the hyperparameters of each model. Under such a consideration, it was observed that Decision Tree correctly predicted the majority of botnet samples with 100% probability, except for the Blue botnet that was classified as a Zeus with a probability of 74.840%. Random Forest obtained similar results for the majority of botnet specimens, except for Blue as mentioned before. In addition, the samples that were correctly classified exposed probability variations on the analyzed specimens. An example of this was found with Random Forest, which initially classified Citadel with a 100% rate, but the GS optimization slightly decreased the hit rate to 98.690%. Similar to the Random Forest model, the K-Nearest Neighbors model incorrectly classified the Zyklon botnet as Citadel with 100% probability. Comparing this model with the rest of the classifiers, it was noted that in Table 5 the K-Nearest Neighbors model before and after implementing the GS algorithm does not exceed 70% accuracy and predicts the Zyklon botnet with a 53.061% rate. Unlike other classifiers, the Naive Bayes model initially predicted only Citadel and Citadel2 botnets, and when the GS model was analyzed it was observed that Blackout, Blue, Liphyra and Black Energy were correctly predicted with higher probability. Based on this analysis, it can be deduced that Naive Bayes might lead to less predictable results when dealing with botnet detection. Finally, both Support Vector Machine (SVM) models (base implementation and GS optimization) were unable to predict the botnet samples. This model exhibited the same unpredictability issues as Naïve Bayes. Lower hit rates are associated with the scarcity of samples on certain botnet specimens.

Furthermore, the prediction results when analyzing the minority and majority classes separately are shown in Table 12 and Table 13, respectively. There, Random Forest, Decision Tree and, K-Nearest Neighbors correctly predicted all botnet types with a high success rate each, and Support Vector Machine (SVM) performed a proper classification as well. On the contrary, Naïve Bayes model was initially not correctly predicting five botnet specimens. Blackout was mistakenly classified as Liphyra 62.620% certainty, and Liphyra, Black Energy, and Zylon were incorrectly classified as Blue. Similarly, Zeus was classified as Citadel with a 99.900% certainty. These results notably improved after applying the Grid Search algorithm, where the model failed on predicting only two botnet types. Zeus was classified as Citadel with a high hit rate and Zyklon was wrongly classified as Black Energy with a 99.500% certainty. Once again, Naive Bayes behaves unexpectedly in the assessed scenarios.

## 5. Discussion

Our experimental validation using GS has shown a substantial improvement in the prediction of the different botnet specimens even in highly unbalanced classes compared to the results shown by Alenazi et al. [26] and Zhou and Pezaros [25] in their previous proposals. In both research works, it was not possible to grasp important considerations regarding the evaluation of the algorithms and the optimization of their hyper parameters.

When analyzing the original CIC-AWS-2018 dataset, the results showed that the Random Forest, Decision Tree and K-Neighbors models yield a classification accuracy close to 100%, similar to the results exposed by Zhou and Pezaros [25], with the difference that our Naive Bayes implementation obtained 64.994%, which, albeit not close to the highest ones, was more accurate than the results obtained by the same authors (52%). Although Zhou and Pezaros [25] achieved high accuracy rates, it was not possible to conclude whether their results were caused by an overfit. However, our approach underscores that by filtering the CIC-AWS-2018 dataset by TCP protocol was possible to counteract the overfitting due to the higher presence of bot samples associated to this transport protocol and using the Grid Search algorithm the hyperparameters of each model were optimized. It was observed at the same time that the Random Forest, Decision Tree and K-Neighbors models attained considerably higher precision compared to the Naive Bayes and Support Vector Machine models, which could not exceed 87% precision when detecting a botnet. Moreover, Random Forest, Decision Tree and K-Neighbors models correctly predicted bots and benign samples with higher hit rates in comparison to Naïve Bayes and SVM. When assessing the model performance, the difference between the classifiers is that Decision Trees and Random Forest models, with high accuracy and precision rates, were also capable of analyzing large amounts of data in less time and thus surpassing the other classifiers.

On the other hand, a detailed evaluation was performed for each specimen of the ISOT HTTP Botnet dataset in terms of accuracy, precision and recall compared with the work of Alenazi et al. [26]. Although in the research conducted by Alenazi et al. [26] the performance of the Random Forest and Decision Tree models outperformed the Naive Bayes model, it was not performed an in-depth inspection on the predictive capabilities of each model. After using the Grid Search algorithm to optimize the hyperparameters, their results showed significant improvements in accuracy, but low recall rates were observed when predicting specimens with few samples. On the opposite, obtaining higher precision rates when evaluating specimens with more samples, so it is inferred they are working with an unbalanced dataset. Bearing this in mind, our approach emphasizes the importance of dividing the dataset into minority and majority classes, thus modifying the “class_weight” parameter in Random Forest, Decision Tree and SVM models, both at base training and with GS. By doing so, our models were able to correctly predict the different botnet specimens (both minority and majority botnet classes) excepting the Naive Bayes model, which failed to predict the Zeus and Zyklon botnets. Likewise, it was observed that the Random Forest and Decision Tree models shown the highest accuracy and precision rates for detecting the minority and majority classes; however, the Naive Bayes failed on detecting the majority classes. As it was shown in the previous performance analysis, Decision Tree and Random Forest could analyze large amounts of data with less processing overhead, hence outperforming the other classifiers when building their models.

As shown by the results, the selection of the most relevant features played an important role. A decision grounded on previous research works where the behavioral metrics disclosed communication patterns for each botnet type in real testing environments. An important fact related to the creation of the CSE-CIC-IDS2018 dataset and the ISOT HTTP Botnet Dataset is that machine learning techniques had already been used to extract the most relevant malware behavioral features from the traffic captured in the network. For all the different types of attacks, the most relevant behavioral metrics for botnet detection have been considered grounded on the research works of Gonzalez-Cuautle et al. [16] and Sharafaldin et al. [41], who have enumerated the most relevant characteristics for training the models. In our research, two additional characteristics were included by analyzing the feature_importances criterion in order to improve the precision of the Random Forest algorithm. Besides such considerations, it was worth comparing the accuracy obtained by the default configuration of the classifiers, so that the application of Grid Search allowed to find the most optimal hyperparameters, which notably contributed to boost up a more accurate detection based on the observed results.

Finally, in the light of the obtained results, it can be concluded that Random Forest and Decision Tree models are generally more appropriate for detecting botnets by classifying large amounts of samples and still performing efficiently. The capabilities of such models demonstrate their suitability to develop defensive countermeasures against malware, a claim reasserted by this research after a deepen analysis. On the opposite, it was found that Naive Bayes showed mostly inaccurate results, a fact explained by the “Naïve assumption” where all the features included in the modeling process are independent from each other, thus improperly describing the behavior of botnets in the aftermath.

## 6. Conclusions

Throughout this research, the state of the art on botnet detection has been thoroughly analyzed in order to understand the characterization of these network threats, their modus operandi, and special attention has been put on identifying the most relevant supervised detection methods. It was intended to set a comparative baseline both at evaluation methodology and classification accuracy, without overlooking any consideration regarding the models parameterization, training and validation of the detection models, without overlooking any evaluation criteria. For our analysis, it have been considered diverse machine-learning approaches to detect different botnet specimens. In order to set up an evaluation baseline, there have been selected two botnet reference datasets have been examined in detail—CIC-AWS-2018 and ISOT HTTP Botnet, both containing behavioral metrics that have led to perform traffic flow analysis. The former with two bot samples (Zeus and Ares) and the latter with eight botnet specimens. Feature selection has been guided by the contributions of similar research works which led to distinguishing two broader feature categories—descriptive and behavioral. Training and validation of the selected machine learning models have been addressed to benchmark the overall classification accuracy. TThe issue of unbalanced datasets has been considered, which led to differentiation of the analysis guided by majority and minority classes. In addition, the Grid Search algorithm was used to optimize its hyperparameters, which has introduced significant improvements in the classification by adjusting different supervised learning algorithms. As suggested by the proposed methodology, an in-depth evaluation was carried out stressing the comparative analysis of traffic flow patterns. It poses the main contribution of our research towards the state of the art in the area of botnet detection. Broadly speaking, it has been shown that Random Forest and Decision Tree models outperformed the rest of the machine learning models. In contrast, Naive Bayes showed the lowest performance based on the overall accuracy. Therefore, it is shown that it is possible to infer the detection of botnets from behavioral patterns. When measuring the execution time, it can be seen that the Support Vector Machines model still poses the main drawbacks in terms of resource consumption, which has been considerably higher than the rest of the classifiers.

In the light of the extensive evaluation performed, the suitability of machine learning for botnet recognition has been proven in this paper. This research supports the outcomes of similar research works based on machine learning for detecting different botnet specimens. Although it has been clear that the proper calibration and training of the machine learning models directly influences their precision rates, their adequacy is yet to be validated with more botnet specimens. Future research outcomes will be focused on extending the evaluation methodology to make a more robust and exhaustive comparison with further supervised deep learning approaches. It is also intended to expand the range of botnet families to be analyzed. Furthermore, the methodology shall be extended to other botnet datasets as well as their real-time evaluation in network testbeds.

## Figures and Tables

**Table 1:** Classification features used in CIC-AWS-2018 dataset and ISOT HTTP Botnet Dataset.

Type	Feature Name	Description
Descriptive metrics	Label (Output)	The class that is assigned to the different malware samples.
	Src_Port	Source port number
	Dst Port	Destination port number
	Protocol	Transport protocol
Behavioural metrics	Flow_Duration	Flow duration in microseconds
	Tot_Fwd_Pkts	Total number of packets transmitting in forward direction
	Tot_Bwd_Pkts	Total number of packets transmitting in backward direction
	TotLen_Bwd_Pkts	Total size of packets transmitting in forward direction.
	TotLen_Fwd_Pkts	Total size of packets transmitting in backward direction.
	Fwd Pkt Len Max	Maximum packet size in the forward direction
	Fwd Pkt Len Min	Mínimum packet size in the forward direction
	Fwd_Pkt_Len_Mean	Average size of packet transmitting in forward direction.
	Bwd_Pkt_Len_Mean	Average size of packet transmitting in backward direction.
	Flow_Byts/s	Number of bytes per second.
	Flow_Pkts/s	Number of flow packets per second
	Fwd_Pkts/s	Flow packet rate transferred in forward direction per second
	Bwd_Pkts/s	Flow packet rate transferred in backward direction per second
	Subflow_Fwd_Byts	Average number of bytes in a subflow in forward direction.
	Subflow_Bwd_Byts	Average number of bytes in a subflow in backward direction.
	Subflow_Bwd_Pkts	Average number of packets in a subflow in forward direction.
	Subflow_Fwd_Pkts	Average number of packets in a subflow in backward direction.

**Table 2:** ML models + GS-based hyperparameters (λ).

ML Models	λ	CIC-AWS-2018 (TCP)	ISOT HTTP Dataset		
			Original	Minority Class	Majority Class
KNN	Algorithm used to compute nearest neighbors	KD tree	Brute	KD tree	Ball Tree
	Number of neighbors to use	2	1	1	1
	Leaf size	30	30	30	30
	Weight function used in prediction	Uniform	Uniform	Uniform	Uniform
SVM	Regularization parameter C	10	10	10	10
	Estimated class weights for unbalanced datasets			Balanced	Balanced
	Penalty	l2	l2	l2	l2
	multi-class strategy	One-vs-rest	One-vs-rest	One-vs-rest	One-vs-rest
	Select the algorithm to either solve the dual or primal optimization problem	False	False	False	False
	Tolerance for stopping criteria	$1\times 10^{-20}$	$1\times 10^{-20}$	$1\times 10^{-20}$	$1\times 10^{-20}$
	Maximum number of iterations to be run	12,000	12,000	12,000	12,000
	Kernel type to be used in algorithm	Linear	Linear	Linear	Linear
DT	Maximum tree depth	20	35	20	35
	Estimated class weights for unbalanced datasets			Balanced	Balanced
	Number of features for best split	19	20	20	20
	Function to measure split quality	GINI	GINI	GINI	GINI
	Strategy used to choose split at each node	Best	Best	Best	Best
	Min. Number of samples required to be at leaf node	2	10	1	20
	Min. Number of samples required to split	10	10	2	10
RF	Use bootstrap samples when building trees	True	True	True	True
	Estimated class weights for unbalanced datasets			Balanced	Balanced
	Function to measure split quality	GINI	GINI	GINI	GINI
	Maximum tree depth	22	10	35	35
	Number of features for best split	19	20	20	20
	Min. Number of samples required to be at leaf node	10	2	1	1
	Min. Number of samples required to split	10	2	2	10
	Number of trees in forest	12	25	12	12
NGB	Smoothing variable	$1\times 10^{-20}$	$1\times 10^{-10}$	$1\times 10^{-20}$	$1\times 10^{-20}$

**Table 3:** Comparative analysis of ML models on the CIC-AWS-2018 dataset filtered by Transmission Control Protocol (TCP) (A).

Metric	Type	Random Forest	Random Forest + GS	Decision Tree	Decision Tree + GS	K-nearest Neighbors	K-nearest Neighbors + GS
Precision	Benign	99.998%	99.998%	99.998%	99.998%	99.985%	99.986%
	Bot (Zeus and Ares)	100.00%	100.00%	100.00%	100.00%	99.982%	99.979%
Recall	Benign	100.00%	100.00%	100.00%	100.00%	99.991%	99.989%
	Bot (Zeus and Ares)	99.996%	99.996%	99.996%	99.996%	99.972%	99.974%
	Accuracy	99.999%	99.999%	99.999%	99.999%	99.984%	99.984%

**Table 4:** Comparative analysis of ML models on the CIC-AWS-2018 dataset filtered by TCP (B).

Metric	Type	Gaussian Naïve Bayes	Gaussian Naïve Bayes + GS	Support Vector Machines (SVM)	Support Vector Machines (SVM) + GS
Precision	Benign	99.962%	99.025%	99.940%	99.943%
	Bot (Zeus and Ares)	77.855%	79.780%	86.972%	86.904%
Recall	Benign	85.197%	87.019%	92.143%	92.186%
	Bot (Zeus and Ares)	99.939%	98.355%	99.895%	99.898%
	Accuracy	90.245%	90.901%	94.812%	94.821%

**Table 5:** Comparative analysis of ML models in the ISOT HTTP Botnet dataset (A).

Metric	Botnet	Random Forest	Random Forest + GS	Decision Tree	Decision Tree + GS	K-Nearest Neighbors	K-Nearest Neighbors + GS
Precision	Blackout	100.000%	100.000%	100.000%	100.000%	93.019%	93.019%
	Blue	47.069%	74.235%	39.230%	74.510%	35.771%	35.696%
	Liphyra	33.993%	100.000%	18.992%	100.000%	13.729%	13.729%
	Black Energy	96.982%	98.725%	97.135%	99.236%	91.174%	91.174%
	Zeus	82.626%	81.948%	83.255%	81.967%	74.941%	74.936%
	Zyklon	94.595%	100.000%	88.136%	97.938%	53.061%	53.061%
	Citadel	54.314%	57.923%	53.342%	58.855%	51.930%	51.980%
	Citadel2	79.709%	81.212%	79.319%	79.116%	73.756%	73.831%
Recall	Blackout	100.000%	99.916%	100.000%	99.916%	82.857%	82.857%
	Blue	29.105%	21.524%	38.055%	21.080%	36.538%	36.501%
	Liphyra	16.694%	11.994%	18.314%	11.994%	13.128%	13.128%
	Black Energy	96.302%	96.419%	96.341%	96.030%	86.454%	86.454%
	Zeus	90.594%	97.376%	83.553%	97.017%	77.337%	77.337%
	Zyklon	81.395%	79.845%	80.620%	73.643%	20.155%	20.155%
	Citadel	54.355%	57.114%	55.702%	49.455%	51.956%	52.033%
	Citadel2	79.832%	81.346%	78.288%	84.183%	73.248%	73.293%
Accuracy		76.604%	79.115%	74.696%	78.893%	69.025%	69.054%

**Table 6:** Comparative analysis of ML models in the ISOT HTTP Botnet dataset (B).

Metric	Botnet	Gaussian Naive Bayes	Gaussian Naive Bayes + GS	Support Vector Machine (SVM)	Support Vector Machine (SVM) + GS
Precision	Blackout	4.792%	100.000%	100.000%	100.000%
	Blue	9.030%	28.155%	0.000%	0.000%
	Liphyra	2.665%	4.048%	0.000%	0.000%
	BlackEnergy	6.061%	100.000%	100.000%	100.000%
	Zeus	20.000%	69.687%	72.822%	72.817%
	Zyklon	0.431%	0.130%	0.000%	0.000%
	Citadel	30.620%	2.941%	51.473%	51.473%
	Citadel2	60.610%	93.803%	65.014%	65.014%
Recall	Blackout	1.261%	99.916%	99.916%	99.916%
	Blue	33.876%	99.408%	0.000%	0.000%
	Liphyra	70.178%	99.514%	0.000%	0.000%
	BlackEnergy	0.156%	91.787%	91.903%	91.865%
	Zeus	0.115%	49.183%	87.116%	87.116%
	Zyklon	0.775%	0.775%	0.000%	0.000%
	Citadel	7.094%	0.192%	4.708%	4.708%
	Citadel2	60.803%	44.917%	91.008%	91.008%
Accuracy		28.812%	46.389%	70.014%	70.012%

**Table 7:** Comparative analysis of minority and majority classes on the ISOT HTTP Botnet Dataset.

DS Division	Metric	Botnet	Random Forest + GS	Decision Tree + GS	K-Nearest Neighbors + GS	Gaussian Naïve Bayes + GS	Support Vector Machine + GS
Minority Classes	Precision	Blackout	100.000%	99.918%	89.426%	100.000%	100.000%
		Blue	99.963%	99.963%	91.844%	100.000%	99.925%
		Liphyra	100.000%	100.000%	80.692%	100.000%	100.000%
		Black Energy	99.883%	99.883%	94.065%	95.784%	98.522%
		Zyklon	100.000%	98.230%	56.061%	15.152%	75.194%
	Recall	Blackout	99.918%	99.918%	89.426%	99.836%	99.836%
		Blue	100.000%	99.963%	95.034%	99.216%	99.216%
		Liphyra	100.000%	100.000%	75.085%	99.831%	100.000%
		Black Energy	100.000%	99.922%	93.918%	94.776%	98.752%
		Zyklon	97.368%	97.368%	32.456%	21.930%	85.088%
	Accuracy		99.944%	99.902%	91.042%	96.554%	98.995%
Majority Classes	Precision	Zeus	94.811%	94.834%	87.478%	71.367%	88.972%
		Citadel	52.542%	50.973%	53.525%	32.890%	48.898%
		Citadel2	88.973%	93.050%	75.854%	95.242%	92.074%
	Recall	Zeus	97.927%	98.268%	88.975%	49.297%	94.164%
		Citadel	79.055%	88.244%	54.173%	97.012%	96.946%
		Citadel2	69.977%	65.007%	74.599%	45.763%	54.157%
	Accuracy		81.044%	80.437%	75.731%	56.266%	75.413%

**Table 8:** Comparison between the CIC-AWS-2018 and ISOT HTTP Botnet detection models with GS.

Dataset	ML Detection Model	Accuracy
Dataset CIC-AWS-2018 (filtered by TCP)	Random Forest	99.999%
	Decision Tree	99.999%
	K-nearest neighbors	99.984%
	Gaussian Naïve Bayes	90.901%
	Support Vector Machine (SVM)	94.821%
ISOT HTTP Botnet Dataset	Random Forest	79.115%
	Decision Tree	78.893%
	K-nearest neighbours	69.054%
	Gaussian Naïve Bayes	46.389%
	Support Vector Machines (SVM)	70.012%
ISOT HTTP Botnet Dataset	Random Forest	99.944%
	Decision Tree	99.902%
	K-nearest neighbours	91.042%
	Gaussian Naïve Bayes	96.554%
	Support Vector Machines (SVM)	98.995%
ISOT HTTP Botnet Dataset	Random Forest	81.044%
	Decision Tree	80.437%
	K-nearest neighbours	75.731%
	Gaussian Naïve Bayes	56.266%
	Support Vector Machines (SVM)	75.413%

**Table 9:** Average accuracy of the different models in both datasets.

Configuration Type	ML Model	CIC-AWS-2018 (TCP)	ISOT HTTP Botnet		
			Original	Minority Class	Majority Class
Base	Random Forest	99.999%	76.737%	99.909%	80.976%
	Decision Tree	99.999%	74.705%	99.916%	80.388%
	K-Nearest Neighbors	99.984%	68.336%	88.934%	74.768%
	Gaussian Naïve Bayes	90.236%	28.563%	29.919%	46.877%
	Support Vector Machine	94.843%	69.864%	97.624%	75.209%
Grid Search	Random Forest	99.998%	79.199%	99.909%	81.073%
	Decision Tree	99.998%	79.077%	99.916%	80.620%
	K-Nearest Neighbors	99.983%	68.340%	88.934%	74.765%
	Gaussian Naïve Bayes	90.893%	46.483%	96.557%	56.462%
	Support Vector Machine	94.797%	69.864%	97.711%	75.695%

**Table 10:** Prediction results using the CIC-AWS-2018 dataset filtered by TCP.

Detection Models	Without GS		With GS		Expected Class
	Predicted Class	Success Probability	Predicted Class	Success Probability	
Random Forest	Benign	100.00%	Benign	100.00%	Benign
Decision Tree	Benign	100.00%	Benign	100.00%	
KNN	Benign	100.00%	Benign	100.00%	
Naive Bayes	Benign	100.00%	Benign	100.00%	
SVM	Benign	100.00%	Benign	100.00%	
Random Forest	Bot	100.00%	Bot	100.00%	Bot
Decision Tree	Bot	100.00%	Bot	100.00%	
KNN	Bot	100.00%	Bot	100.00%	
Naive Bayes	Bot	100.00%	**Benign**	**100.00%**	
SVM	**Benign**	**64.850%**	**Benign**	**64.680%**	

**Table 11:** Prediction results using ISOT HTTP Botnet Dataset.

Detection Model	Without GS		With GS		Expected Class
	Predicted Class	Success Probability	Predicted Class	Success Probability	
Random Forest	Blackout	100.000%	Blackout	100.000%	Blackout
Decision Tree	Blackout	100.000%	Blackout	100.000%	
KNN	Blackout	100.000%	Blackout	100.000%	
Naive Bayes	**Liphyra**	**69.210%**	Blackout	100.000%	
SVM	Blackout	43.670%	Blackout	43.670%	
Random Forest	Blue	51.770%	**Zeus**	**74.420%**	Blue
Decision Tree	Blue	100.000%	**Zeus**	**74.840%**	
KNN	Blue	100.000%	Blue	100.000%	
Naive Bayes	**Liphyra**	**59.490%**	Blue	100.000%	
SVM	**Zeus**	**59.410%**	**Zeus**	59.410%	
Random Forest	Liphyra	100.000%	Liphyra	100.000%	Liphyra
Decision Tree	Liphyra	100.000%	Liphyra	100.000%	
KNN	Liphyra	100.000%	Liphyra	100.000%	
Naive Bayes	**Blue**	**52.190%**	Liphyra	100.000%	
SVM	**Citadel2**	48.670%	**Citadel2**	**48.670%**	
Random Forest	Black Energy	100.000%	Black Energy	100.000%	Black Energy
Decision Tree	Black Energy	100.000%	Black Energy	100.000%	
KNN	Black Energy	100.000%	Black Energy	100.000%	
Naive Bayes	**Citadel**	**44.770%**	Black Energy	99.920%	
SVM	Black Energy	54.400%	Black Energy	54.400%	
Random Forest	Zeus	84.290%	Zeus	74.600%	Zeus
Decision Tree	Zeus	100.000%	Zeus	74.840%	
KNN	Zeus	100.000%	Zeus	100.000%	
Naive Bayes	**Liphyra**	**60.660%**	**Blue**	**100.000%**	
SVM	Zeus	60.050%	Zeus	60.050%	
Random Forest	Zyklon	100.000%	Zyklon	100.000%	Zyklon
Decision Tree	Zyklon	100.000%	Zyklon	100.000%	
KNN	**Citadel**	**100.00%**	**Citadel**	**100.00%**	
Naive Bayes	**Blue**	**56.840%**	**Citadel**	**99.980%**	
SVM	**Zeus**	**50.130%**	**Zeus**	50.130%	
Random Forest	Citadel	100.000%	Citadel	98.690%	Citadel
Decision Tree	Citadel	100.000%	Citadel	99.550%	
KNN	Citadel	100.000%	Citadel	100.000%	
Naive Bayes	Citadel	91.950%	**Liphyra**	**100.000%**	
SVM	**Citadel2**	**45.779%**	**Citadel2**	45.779%	
Random Forest	Citadel2	100.000%	Citadel2	100.000%	Citadel2
Decision Tree	Citadel2	100.000%	Citadel2	100.000%	
KNN	Citadel2	100.000%	Citadel2	100.000%	
Naive Bayes	Citadel2	74.860%	**Zyklon**	**99.940%**	
SVM	Citadel2	88.030%	Citadel2	88.030%	

**Table 12:** Prediction results using minority classes included on the ISOT HTTP Botnet Dataset.

Detection Model	Without GS		With GS		Expected Class
	Predicted Class	Success Probability	Predicted Class	Success Probability	
Random Forest	Blackout	100.000%	Blackout	100.000%	Blackout
Decision Tree	Blackout	100.000%	Blackout	100.000%	
KNN	Blackout	100.000%	Blackout	100.000%	
Naive Bayes	**Liphyra**	**62.620%**	Blackout	100.000%	
SVM	Blackout	70.700%	Blackout	71.460%	
Random Forest	Blue	100.000%	Blue	100.000%	Blue
Decision Tree	Blue	100.000%	Blue	100.000%	
KNN	Blue	100.000%	Blue	100.000%	
Naive Bayes	Blue	50.530%	Blue	100.000%	
SVM	Blue	96.690%	Blue	96.760%	
Random Forest	Liphyra	100.000%	Liphyra	100.000%	Liphyra
Decision Tree	Liphyra	100.000%	Liphyra	100.000%	
KNN	Liphyra	100.000%	Liphyra	100.000%	
Naive Bayes	**Blue**	**60.630%**	Liphyra	100.000%	
SVM	Liphyra	94.780%	Liphyra	94.040%	
Random Forest	Black Energy	100.000%	Black Energy	100.000%	Black Energy
Decision Tree	Black Energy	100.000%	Black Energy	100.000%	
KNN	Black Energy	100.000%	Black Energy	100.000%	
Naive Bayes	**Blue**	**72.350%**	Black Energy	100.000%	
SVM	Black Energy	72.740%	Black Energy	72.980%	
Random Forest	Zyklon	100.000%	Zyklon	100.000%	Zyklon
Decision Tree	Zyklon	100.000%	Zyklon	100.000%	
KNN	Zyklon	100.000%	Zyklon	100.000%	
Naive Bayes	**Blue**	**69.090%**	**Black Energy**	**99.500%**	
SVM	Zyklon	95.320%	Zyklon	95.520%	

**Table 13:** Prediction results using majority classes included on the ISOT HTTP Botnet Dataset.

Detection Model	Without GS		With GS		Expected Class
	Predicted Class	Success Probability	Predicted Class	Success Probability	
Random Forest	Zeus	100.000%	Zeus	100.000%	Zeus
Decision Tree	Zeus	100.000%	Zeus	100.000%	
KNN	Zeus	100.000%	Zeus	100.000%	
Naive Bayes	**Citadel**	**99.900%**	**Citadel**	**100.000%**	
SVM	Zeus	93.260%	Zeus	92.810%	
Random Forest	Citadel	100.000%	Citadel	100.000%	Citadel
Decision Tree	Citadel	100.000%	Citadel	100.000%	
KNN	Citadel	100.000%	Citadel	100.000%	
Naive Bayes	Citadel	91.950%	Citadel	100.000%	
SVM	Citadel	52.020%	Citadel	52.510%	
Random Forest	Citadel2	100.000%	Citadel2	100.000%	Citadel2
Decision Tree	Citadel2	100.000%	Citadel2	100.000%	
KNN	Citadel2	100.000%	Citadel2	100.000%	
Naive Bayes	Citadel2	74.860%	Citadel2	99.080%	
SVM	Citadel2	82.500%	Citadel2	80.140%	

## Appendix A. Initial Hyperparameters

**Table A1:** Algorithms with Their Initial Hyperparameters λ.

Detection Model	λ	CIC-AWS-2018		ISOT HTTP Botnet	
		Original	Filtered by TCP	Original	Minority and Majority Class
KNN	Algorithm used to compute nearest neighbors	KD tree	KD tree	KD tree	KD tree
	Number of neighbors to use	2	2	1	1
	Leaf size	30	30	30	30
	Weight function used in prediction	Uniform	Uniform	Uniform	Uniform
SVM	Regularization parameter C	175	175	175	175
	Estimate class weights for unbalanced datasets	—	—	—	Balanced
	Penalty	l2	l2	l2	l2
	multi-class strategy	One-vs-rest	One-vs-rest	One-vs-rest	One-vs-rest
	Select the algorithm to either solve the dual or primal optimization problem	False	False	False	False
	Tolerance for stopping criteria	$1\times 10^{-10}$	$1\times 10^{-10}$	$1\times 10^{-10}$	$1\times 10^{-10}$
	Maximum number of iterations to be run	12,000	12,000	12,000	12,000
	Kernel type to be used in algorithm	Linear	Linear	Linear	Linear
DT	Maximum tree depth	51	14	51	35
	Estimate class weights for unbalanced datasets	—	—	—	Balanced
	Number of features for best split	19	19	20	20
	Function to measure split quality	GINI	GINI	GINI	GINI
	Strategy used to choose split at each node	Best	Best	Best	Best
	Min. Number of samples required to be at leaf node	1	1	1	1
	Min. Number of samples required to split	2	2	2	2
RF	Use bootstrap samples when building trees	True	True	True	True
	Estimate class weights for unbalanced datasets	—	—	—	Balanced
	Function to measure split quality	GINI	GINI	GINI	GINI
	Maximum tree depth	35	22	35	35
	Number of features for best split	19	19	20	20
	Min. number of samples required to be at leaf node	1	1	1	1
	Min. number of samples required to split	2	2	2	2
	Number of trees in forest	12	12	12	12
NGB	Smoothing variable	$1\times 10^{-10}$	$1\times 10^{-10}$	$1\times 10^{-10}$	$1\times 10^{-10}$

## Appendix B. Inherent Hyperparameters and Values

**Table A2:** Algorithms with Their Inherent Hyperparameters and Values.

Detection Model	λ	CIC-AWS-2018 Filtered by TCP Using GS	ISOT HTTP Botnet Using GS	
			Original	Minority and Majority Classes
KNN	Algorithm used to compute nearest neighbors	[‘kd_tree’, ‘ball_tree’, ‘brute’]	[‘kd_tree’, ‘ball_tree’, ‘brute’]	[‘kd_tree’, ‘ball_tree’, ‘brute’]
	Metric	minkowski	minkowski	minkowski
	Leaf size	[30,50,80]	[30,50,70]	[30,50,70]
	Number of neighbors to use	[4,10,2]	[4,10,2]	[2,10,1]
SVM	Regularization parameter C	[10,100,175]	[10,100,175]	10
	Estimate class weights for unbalanced datasets	—	—	Balanced
	Penalty parameter	l2	l2	l2
	Tolerance for stopping criteria	[$1\times 10^{-20}$,$1\times 10^{-10}$,$1\times 10^{-5}$]	[$1\times 10^{-20}$,$1\times 10^{-10}$,$1\times 10^{-5}$]	[$1\times 10^{-20}$,$1\times 10^{-10}$]
	Maximum number of iterations to be run	12000	12000	12000
	Kernel type to be used in algorithm	Linear	Linear	Linear
DT	Maximum tree depth	[20,30,14]	[20,30,10]	[20,35,10]
	Estimate class weights for unbalanced datasets	—	—	Balanced
	Number of features for best split	19	20	20
	Min. Number of samples required to be at leaf node	[10,20,2]	[10,20,2]	[10,20,1]
	Min. Number of samples required to split	[10,20,2]	[10,20,2]	[10,20,2]
RF	Maximum tree depth	[22,51,10]	[20,50,10]	[20,35,10]
	Estimate class weights for unbalanced datasets	—	—	Balanced
	Number of features for best split	19	20	20
	Min. Number of samples required to be at leaf node.	[10,20,30]	[10,20,30]	[10,20,1]
	Min. Number of samples required to split	[10,20,30]	[10,20,30]	[10,20,2]
	Number of trees in forest	25	12	12
NBG	Smoothing Variable	[$1\times 10^{-20}$,$1\times 10^{-10}$,$1\times 10^{-5}$]	[$1\times 10^{-20}$,$1\times 10^{-10}$,$1\times 10^{-5}$]	[$1\times 10^{-20}$,$1\times 10^{-10}$,$1\times 10^{-5}$]
